# Effects of Resistance Exercise on Glycated Hemoglobin and Functional Performance in Older Patients with Comorbid Diabetes Mellitus and Knee Osteoarthritis: A Randomized Trial

**DOI:** 10.3390/ijerph17010224

**Published:** 2019-12-27

**Authors:** Shu-Mei Chen, Feng-Chih Shen, Jung-Fu Chen, Wen-Dien Chang, Nai-Jen Chang

**Affiliations:** 1 Department of Sports Medicine, Kaohsiung Medical University, Kaohsiung 807, Taiwan; may05010421@gmail.com; 2 Division of Endocrinology & Metabolism, Department of Internal Medicine, Kaohsiung Chang Gung Hospital, Chang Gung University College of Medicine, Kaohsiung 833, Taiwan; carcinom@cgmh.org.tw (F.-C.S.); 0722cjf@cgmh.org.tw (J.-F.C.); 3 Department of Sport Performance, National Taiwan University of Sport, Taichung 404, Taiwan; changwendien@ntupes.edu.tw; 4 Ph.D. Program in Biomedical Engineering, Kaohsiung Medical University, Kaohsiung 807, Taiwan; 5 Regenerative Medicine and Cell Therapy Research Center, Kaohsiung Medical University, Kaohsiung 807, Taiwan

**Keywords:** therapeutic exercise, physical activity, diabetes, performance, osteoarthritis

## Abstract

Type 2 diabetes mellitus (T2DM) is significantly associated with osteoarthritis (OA). This study investigated the effects of two resistance exercise approaches on glycated hemoglobin (HbA1c) level and function performance. Enrolled were 70 older patients with both T2DM and knee OA. The dynamic group performed resistance exercises with an elastic resistance band. The isometric group underwent isometric contraction exercises. After the 12-week intervention, a significant within-group improvement (all *p* < 0.001) was observed for the chair stand test (CST; 10.8%, vs. 7.1%), timed up and go (TUG) test (12.6% vs. 7.6%), Western Ontario and McMaster Universities Osteoarthritis (WOMAC) physical function subscale (62.3% vs. 36.1%), and overall WOMAC (54.5% vs. 34.5%) in the dynamic and isometric group, respectively. In addition, in terms of between-group differences, the dynamic group had significant improvements in CST (*p* = 0.011), TUG (*p* < 0.001), WOMAC physical function subscale (*p* = 0.033), and overall WOMAC (*p* = 0.036) scores compared with the isometric group. However, no significant change in HbA1c was observed in either group. In conclusion, the dynamic resistance exercise significantly improved muscle strength, dynamic balance, and physical function in this comorbid population; however, there was no notable difference in change in HbA1c among different resistance exercises.

## 1. Introduction

Type 2 diabetes mellitus (T2DM) is a major global health burden that results in other complications, such as physical disability, neuropathy, cardiovascular disease, and chronic comorbidity [1]. Exercise is an important non-pharmacological intervention for treating T2DM [2]. The benefit of exercise is associated with the capability of muscle contractile activity to augment insulin sensitivity, reduce insulin resistance, and stimulate glucose uptake and clearance [3], thereby reducing inflammation and promoting physical activity. The American College of Sports Medicine (ACSM) indicated that exercises for older adults suggest including aerobic exercise, resistance exercise, and joint activity [4].

Patients with T2DM show an inflammatory response because of their overweight status or hyperglycaemia, and this can damage the articular cartilage [5]. Therefore, DM might be a predictor of osteoarthritis (OA). A previous review and meta-analysis reported that the prevalence of DM was 14.4% in patients with OA; by contrast, the prevalence of OA was 29.5% in patients with DM [6]. Another systematic review and meta-analysis discovered a significant association between T2DM and the development or presence of radiographic and symptomatic OA (odds ratio (OR): 1.21, 95% confidence interval (CI): 1.02–1.41) [7].

OA results in dysfunction, and reduces independence in performing daily activities [8]. Lower extremity muscle weakness, particularly in the quadriceps and gluteus muscles, was associated with radiographic knee OA and increased disability in patients with OA [9]. According to a review published in the Cochrane Database of Systematic Reviews, an exercise program can reduce pain and effectively improve the physical function of patients with knee OA [10]. Regarding strength training mode, interventions that use either dynamic or isometric resistance exercise positively impact the symptoms of OA [11]. A dynamic exercise is essential to move through full range of motion; the whole length of the target muscle is stimulated. Contrary to the dynamic exercise, the isometric exercise creates no change in the length of the muscle. Thus far, which means is superior in changes of strength and physical function remains unclear. Jan et al. used leg press machines as a dynamic exercise to train patients with knee OA for 8 weeks, with higher and lower intensity groups having one-repetition (rep) maximum (RM) of 60%–80% and 10%–30%, respectively [12]. After the intervention, both groups showed a significantly increased strength of the knee extensors and flexors. Although the use of machines for exercise training is beneficial for patients with knee OA, conventional resistance-training equipment is not cheap, portable, or easy to use and requires supervision in case of sports injury. Therefore, as an alternative, the use of an elastic band as a dynamic exercise in clinical rehabilitation [13], and home-based exercise programs [14] has emerged.

Studies have reported the positive effects of resistance training on patients with OA or with T2DM [15,16]. However, it is not known whether dynamic resistance exercises are superior to isometric resistance exercises in older patients with comorbid T2DM and knee OA when delivered through a home-based rehabilitation program, particularly in this comorbid population. This comparative effectiveness study investigated the effects of these two resistance exercise approaches on glycated hemoglobin (HbA1c) level in older patients with comorbid T2DM and knee OA. Concurrently, exercises were prescribed for these patients to improve their muscle strength (ie 30-s chair stand test (CST)), dynamic balance (ie 3-m timed up and go (TUG) test), and physical function (the Western Ontario and McMaster Universities Osteoarthritis scale (WOMAC) score). We hypothesized that dynamic resistance exercise would be the most effective in improving muscle strength, dynamic balance, and physical function in older patients with comorbid T2DM and knee OA; changes in HbA1c level were comparable among different resistance exercise approaches.

## 2. Materials and Methods

### 2.1. Study Design

This study was approved by the Institutional Review Board of Chang Gung Medical Foundation. This study protocol was registered with ClinicalTrials.gov (NCT04026139). Regarding recruitment, participants with available electronic medical records from visits to Kaohsiung Chang Gung Memorial Hospital in Taiwan were targeted. Clinical staff took the initiative to approach patients who met the inclusion criteria directly. Patients with acquired T2DM and knee OA treated as outpatients in the Division of Endocrinology and Metabolism of Kaohsiung Chang Gung Medical Hospital were eligible for this study. After explaining the study purpose to eligible patients and obtaining their written informed consent, we randomly allocated them to either the dynamic or isometric group. Random allocations were concealed in opaque, sealed envelopes (A lot: dynamic group; B lot: isometric group) prepared by independent clinical staff members who were not involved in the recruitment. All patients drew one lot by themselves. Patients’ identification was recorded on each lot. All selected patients in both groups were blinded to their grouping, and all the outcome measurements were assessed at the Division of Endocrinology and Metabolism by the same investigator. The HbA1c level, muscle strength, dynamic balance, and physical function of all enrolled patients were examined before and after 12 weeks of intervention.

### 2.2. Participants

Inclusion criteria for this study were as follows: age between 60 and 70 years; comorbid T2DM and knee OA diagnosed by a physician; diagnostic criteria for the diagnosis of T2DM based on the ‘Classification and Diagnosis of Diabetes: Standards of Medical Care in Diabetes’ by the American Diabetes Association [17]; and Kellgren and Lawrence (K&L) grade ≤3 based on the findings of plain radiographs [18]. Exclusion criteria were as follows: inability to perform any single self-activity; T2DM with complications, such as lower extremity neuropathy, retinopathy, stroke, foot wounds, or amputations; K&L grade >3, hip or knee arthroplasty; history of myocardial infarction; uncontrolled liver disease (e.g., liver cirrhosis, hepatocellular carcinoma, and acute liver failure); end-stage renal disease; and malignant hypertension. Patients prescribed medications had their related dosing schedule maintained throughout the investigation. This study was conducted at Kaohsiung Chang Gung Medical Hospital.

### 2.3. Interventions

#### 2.3.1. Dynamic Resistance Exercise Group

Considering that older patients with T2DM and knee OA were enrolled in this study, we intended to design a safe, easy, and convenient elastic band exercise program. The ACSM guideline recommends that strength training exercises should be performed three times a week for older patients. Principle resistance training should involve 8–15 RM, which must include 5–10 min of warm-up and cool-down exercises to prevent muscle soreness, discomfort, and injury [19]. Therefore, in this study, we used an elastic band resistance training program that involved 10 RM + rated perceived exertion (RPE) of 13, with the scale ranging from 6 to 20 [20].

**Figure 1:**
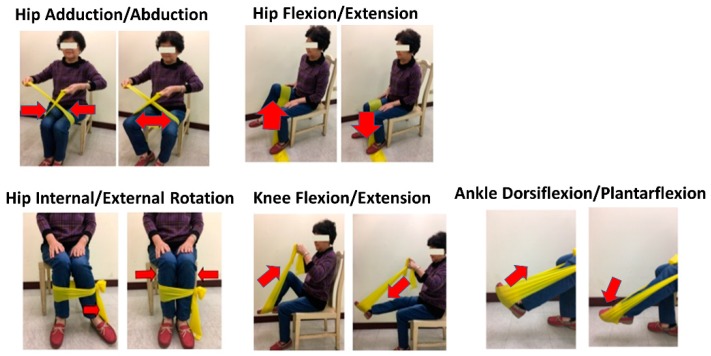
Dynamic exercise programs with elastic bands.

To enable participants to adapt to the resistance training program, exercise instructions were provided to participants by clinical staff in the medical center, and a lighter elastic band was used in the first 2 weeks [21]. Participants’ adherence to the exercise program was tracked every week through communication consistent phone calls or using a communication application based on training sessions. Exercises progressed by adding a greater stretch to the band prescribed in order to provide greater resistance or by moving up to the next difficulty level of resistance band. The 12-week exercise program in this study was developed after making modifications to exercise programs used in previous study [22]. The complexity of the exercise program was adjusted by a physical therapist including of hip joint abduction/adduction, flexion/extension, external/internal rotation, knee joint flexion/extension, and ankle joint plantar/dorsiflexion (Figure 1). In the program, seated, open-chain exercises were used to train the major muscle groups of the lower extremities without challenging balance. In the dynamic group, patients performed 10 reps/set × 5 sets/day × 3 days/week × 12 weeks. To ensure the quality of exercises performed at home, participants and their family members were required to examine whether all prescribed movements were performed correctly in the education room. In addition, they were provided handouts with highlighted notes as reminders. They also received images and a video showing exercise movements on a mobile application.

#### 2.3.2. Isometric Resistance Exercise Group

Exercise movements performed in the isometric group were the same as those performed in the dynamic group, except for use of an elastic band. Each participant was instructed by clinical staff in the medical center to perform active joint range-of-motion exercises with free bodyweight and isometric contraction exercises in the sitting position for 5 sets of 10 reps with a 10-s hold, three times a week, for 12 weeks. Adherence of participants in the isometric group to exercises and physical activity was evaluated through weekly phone calls during the 12-week intervention.

### 2.4. Outcome Measures

#### 2.4.1. HbA1c Analysis

HbA1c is an indicator of the average plasma sugar level in the past three months and is routinely used to monitor patients with T2DM [23]. The HbA1c level was measured using a boronate-affinity chromatography-based high-performance liquid chromatography system with an automated benchtop HbA1c analyser (Premier Hb9210, Bray, Ireland/Kansas City, MO, USA). The normal range for the HbA1c level is between 4% and 5.6%. HbA1c levels between 5.7% and 6.4% indicate that patients have prediabetes. Levels of 6.5% or higher indicate that patients have diabetes.

#### 2.4.2. Chair Stand Test (CST) of 30 Seconds

The 30-s CST is used to assess the muscle strength of the lower extremity in patients with OA. Participants were asked to sit on a standard chair, with arms crossed over the chest and then stand up to a fully extended standing position as many times as possible. The maximum number of reps completed in 30 s was recorded. This test for knee OA has a high intraclass correlation coefficient (ICC) of 0.89, as indicated by the Osteoarthritis Research Society International (OARSI) [24]. In this study, the ICC was 0.964, indicating excellent test–retest reliability. In addition, minimum detectable change (MDC) value was calculated as 1.251 corresponding to the standard error of measurement (SEM) of 0.434.

#### 2.4.3. Timed Up and Go (TUG) Test

The TUG test is used to examine the dynamic balance of patients with musculoskeletal conditions during multiple activities, such as the sit-to-stand movement, walking a short distance, and changing direction while walking. The OARSI has recommended the TUG test as an outcome measure for patients with hip or knee OA [25]. Participants were asked to sit on a standard chair and place their hands on their thighs. Then, when participants heard the command ‘start’ from the investigator (tester started the stopwatch simultaneously), they were asked to begin rising from the chair (with or without using their arms), walk to the target placed 3 m in front of them (running was not allowed), bypass the target, walk back to the chair, and sit down (stopwatch was stopped after a patient sat back in the chair). The time required to perform these activities was recorded in seconds. The TUG test is highly reliable, with an adequate minimum detectable change for clinical use in individuals with knee OA of K&L grades 1–3; the intrarater and interrater reliabilities of the TUG test were 0.97 and 0.96, respectively [26]. The minimal clinically important difference (MCID) was set as a decrease of 1.2 s in the total time required compared with baseline data [27]. In this study, the ICC was 0.968, indicating excellent test-retest reliability. In addition, MDC value was calculated as 0.869 in corresponding to SEM of 0.313.

#### 2.4.4. Western Ontario and McMaster Universities Osteoarthritis (WOMAC) Scale Scores

The WOMAC scale is a highly reliable questionnaire on activities of daily living used for patients with OA [28]. This scale contains three subscales of pain, stiffness, and physical function. A 5-point Likert scale measures responses ranging from 0 (no disability) to 4 (extremely severe disability). Higher scores indicate greater disability. In this study, the ICC was 0.935, indicating excellent test–retest reliability. In addition, MDC value for subscale of pain, stiffness, and physical function, and total scores was calculated as 1.349, 0.579, 2.311, 3.886, respectively.

### 2.5. Statistical Analyses

Sample size was calculated with the TUG test for older patients as the main outcome and was based on an anticipated difference of 1.2 s in the TUG test timing between the groups from baseline to post-intervention [29]; this time change was adopted as the MCID [27,29]. The calculation was also based on an anticipated standard deviation of 1.5 s, an alpha level of 0.05, and a desired power of 80%. A minimum sample size of 26 patients per group was estimated. In addition, assuming a dropout rate of 15% [30], we enrolled at least 30 participants in each group.

Statistical analyses were performed using SPSS, version 22.0 (Chicago, IL, USA). All values are presented as the mean and standard deviation. Patients’ baseline characteristics, namely sex, height, weight, body mass index, duration of T2DM, and discrete variables (severity of knee OA), were tested using the independent-samples *t*-test for continuous data and Fisher’s exact test of independence for categorical data. The significance level (α) was set at *p* < 0.05. Data were tested statistically for normality (Shapiro–Wilk test, *p* > 0.05), and their homogeneity of variance was confirmed using Levene’s test. If the sphericity assumption was violated in Mauchly’s sphericity test, the Greenhouse–Geisser adjustment was adopted to correct the degrees of freedom.

A 2 (time: baseline vs. post-intervention) × 2 (group: dynamic vs. isometric group) repeated measures analysis of variance was performed to examine the effects of different groups on the dependent variables. In the event of a significant group × time interaction, a follow-up was conducted using the t test to determine the effect. For significant main effects of intervention, paired *t*-tests within each group were performed to determine the intervention effects.

To verify that scores on the 30-s CST, TUG test, and WOMAC scores were greater than measurement error, MDC value was calculated. To determine the MDC, SEM was calculated first by the formula:

$$\mathrm{SEM}=\mathrm{SDtest}\times \sqrt{\left(1-\mathrm{ICC}\right)}$$

where SDtest is the standard deviation of scores from the first test and ICC is the test-retest intraclass correlation coefficient. Subsequently, MDC was calculated as $d=1.96\times \sqrt{2}\times \mathrm{SEM}$. The effect size (Cohen’s d) was calculated to show the magnitude of the effects (d = M1 − M2 /σpooled) for each group.

## 3. Results

### 3.1. Flow of Participants through the Study

**Figure 2:**
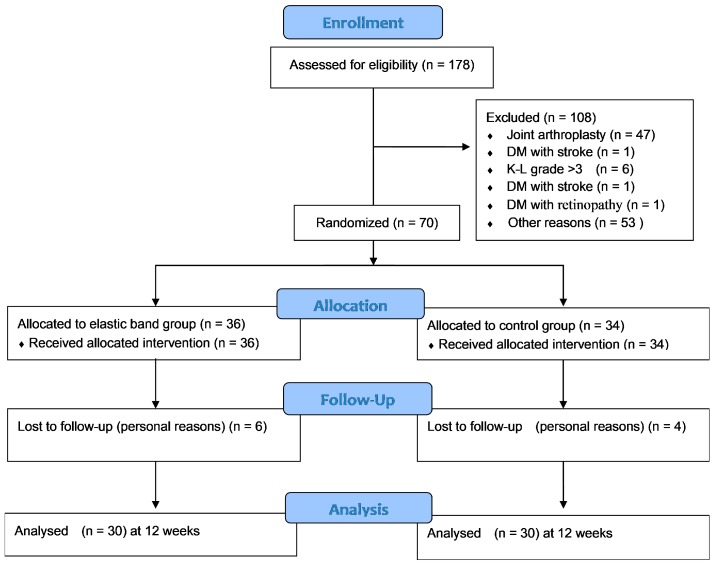
CONSORT flow diagram.

A total of 178 patients with an initial diagnosis of T2DM and knee OA were identified during the study recruitment period. Of them, 108 patients who did not meet the inclusion criteria were excluded. A total of eligible 70 patients were randomly assigned to the dynamic or isometric group. Of these 70 patients, 10 were excluded because of loss of follow-up (personal reasons, did not return, or loss of contact). Finally, 60 participants completed the intervention, including 30 from the dynamic group and 30 from the isometric group. The adherence rate of patients who participated in the intervention was 83% in the dynamic group and 88% in the isometric group. Demographic characteristics, duration of T2DM, HbA1c level, OA grade, or medications were shown between the dynamic and isometric groups (Table 1, Figure 2). No significant differences were observed between the dynamic group and isometric group in the baseline data, including demographic characteristics, duration of T2DM, HbA1c level, OA grade, and CST, TUG, and WOMAC scores (*p* > 0.05) (Table 1 and Table 2). Furthermore, no participants experiencing aggravation of arthritic symptoms and/or had some difficulty in continuing to do the exercise until the end of study.

### 3.2. HbA1c Outcomes

**Figure 3:**
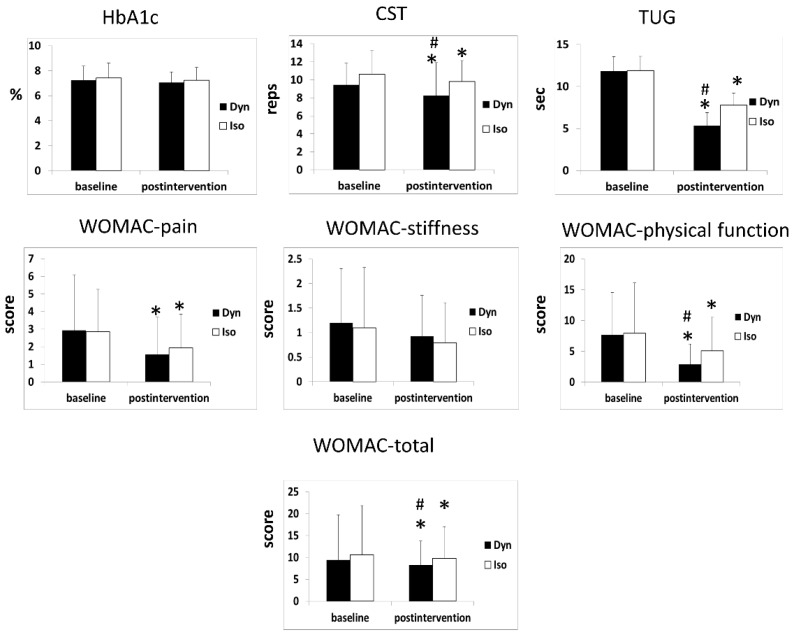
Improvements in HbA1C, CST, TUG, and WOMAC after the 12-week intervention between groups. Dyn = dynamic group, Iso = isometric group. * vs baseline (*p* < 0.05) # vs Iso (*p* < 0.05).

For HbA1c level, the main effects were not statistically significant over time within groups (*p* = 0.167) or between groups (*p* = 0.462). Moreover, no significant time-by-group interaction was noted (*p* = 0.957) (Table 2, Figure 3).

### 3.3. CST 30-s Outcomes

For the 30-s CST, main effects were statistically significant over time within groups (*p* < 0.001) and between groups (*p* = 0.015). No significant time-by-group interactions were observed (*p* = 0.216) (Table 2, Figure 3). In the post hoc test results, the 30-s CST scores of the dynamic group (*p* = 0.004) and isometric group (*p* = 0.004) were significantly better post-intervention than at baseline. Furthermore, the dynamic group demonstrated significant improvement in 30-s CST score (*p* = 0.011) compared with the isometric group (Figure 3).

### 3.4. TUG Test Outcomes

For the TUG test, the main effects were statistically significant over time within groups (*p* < 0.001) and between groups (*p* < 0.001). Moreover, no significant time-by-group interaction was observed (*p* = 0.222; Table 2). In the post hoc test results, the TUG test scores of the dynamic group (*p* < 0.001) and isometric group (*p* = 0.002) were significantly better post-intervention than at baseline. Furthermore, the dynamic group demonstrated a significant improvement in TUG test score (*p* < 0.001) compared with the isometric group (Figure 3). Only the dynamic group reached the MCID.

### 3.5. WOMAC Scores

For the WOMAC subscale of pain, the main effects were statistically significant over time within group (*p* < 0.001). Significant effects were neither found between groups (*p* = 0.374) nor for time-by-group interaction (*p* = 0.374) (Table 2). In the post hoc test results, the WOMAC subscale of pain scores of the dynamic group (*p* = 0.001) and isometric group (*p* = 0.004) were significantly better post-intervention than at baseline.

For the WOMAC subscale of stiffness, statistical significance was not reached for the main effects over time within groups (*p* = 0.131) or between groups (*p* = 0.525) or for the time-by-group interaction (*p* = 0.38) (Table 2).

For the WOMAC subscale of physical function, main effects were statistically significant over time within groups (*p* < 0.001) and between groups (*p* = 0.015). Additionally, a significant time-by-group interaction was noted, indicating that the dynamic group demonstrated significant improvement compared with the isometric group (*p* = 0.035) (Table 2). In the post hoc test results, both groups exhibited significant improvement in scores on the WOMAC subscale of physical function post-intervention compared with baseline values (dynamic group: 62.3%, *p* < 0.001; isometric group: 36.1%, *p* = 0.007; Table 2). Furthermore, the dynamic group demonstrated significant improvement in scores on the WOMAC subscale of physical function (*p* = 0.033) compared with the isometric group (Figure 3).

For the total score of the WOMAC scale, main effects were statistically significant over time within groups (*p* < 0.001) and between groups (*p* = 0.015). Moreover, a significant time-by-group interaction was observed, indicating that the dynamic group demonstrated significant improvement compared with the isometric group (*p* = 0.026) (Table 2). In the post hoc test results, both groups exhibited significant improvement in scores on the WOMAC subscale of physical function post-intervention compared with baseline values (dynamic group: 54.5%, *p* < 0.001; isometric group: 34.5%, *p* = 0.004; Table 2). Furthermore, the dynamic group demonstrated significant improvement in total score on the WOMAC (*p* = 0.036) compared with the isometric group (Figure 3).

## 4. Discussion

This is the first study to investigate the effects of dynamic or isometric resistance training approaches as a home-based rehabilitation is superior in improving HbA1c level, muscle strength, dynamic balance, and functional activities of the lower limbs in patients with comorbid T2DM and mild-to-moderate knee OA. Our results indicated that a 12-week dynamic resistance training program leads to significant improvements on CST, TUG test, and WOMAC scores, but is comparable to an isometric exercise on HbA1c changes.

In terms of HbA1c control, neither the dynamic and isometric groups exhibited any adverse effects (e.g. hypoglycaemia) after the 12-week intervention. This finding may be attributed to the home-based exercise program in which follow-up was conducted through phone calls and for which inexpensive self-directed physical exercises were used to increase participants’ adherence, instead of gym-based exercise programs requiring facilities that can be financially inaccessible for patients [31]. In this study, investigators successfully implemented the home-based program by conducting a follow-up through telephone calls or using a communication application to track participants’ execution of exercises, as indicated by a high adherence rate of >80% in both the groups. Dunstan et al. reported that patients’ adherence, self-execution of exercises, and changes in intensity explain the effectiveness of home-based resistance training in controlling glycaemia [32]. However, we need to investigate that the optimal dose of exercise could impact HbA1c level in the future.

In the present study, after the intervention, the dynamic group showed a significant improvement in the muscle strength of the lower extremities (ie CST) compared with the isometric group (10.8% vs. 7.1%), indicating that home-based elastic band exercises significantly improved the muscle strength of the lower limbs [20]. However, the isometric group also showed improvement in the muscle strength of the lower extremities. The finding may be because the isometric group performed isometric contraction exercises that included multijoint movements instead of being sedentary (no intervention); this finding is similar to that reported by Watanabe et al. [33]. This result indicates that either adding external weight or performing free bodyweight exercises to achieve a postural change in an antigravity condition could increase the muscle strength of older patients [33]. To maintain the benefits of training, it is necessary to continue exercise, similar to the 12-week exercise program adopted in this study.

The WOMAC is usually used to assess pain, stiffness, and physical function in patients with knee OA. The percent change between baseline and post-intervention for the WOMAC total score was significantly higher in the dynamic group (54.5%) than in the isometric group (34.5%). This result may be attributed to the association of exercise with decreased pain, which in turn improves physical activity. A previous study has demonstrated that programs with multiple sets of exercises have a greater effect than programs with a single set of exercises [34]. In line with our study, these exercise programs included hip, knee and ankle joints, and the majority of key muscle groups play a pivot role in muscle strength and physical function. Furthermore, interventional resistance training may activate motor neurons, which promote the reduction of activation around pain nerve fibers or the release of endogenous opioids (such as endorphin and serotonin) from the central nervous system, reducing the feeling of pain [35]. Therefore, pain and physical activity are mutually influential. Specifically, improving physical function is nearly as crucial as alleviating pain. Therefore, we further analysed this relationship and observed significant correlations between pain and WOMAC total scores (*p* < 0.001, r = 0.725) and WOMAC physical function subscale scores (*p* = 0.002, r = 0.536) in the dynamic group. Furthermore, the scores of the WOMAC physical function subscale after treatment were significantly better in the dynamic group than in the isometric group (*p* = 0.033; Table 2, Figure 3).

The main strength of this study is that it revealed the feasibility of enhancing functional performance in older adults with T2DM and knee OA through the provision of instructions by clinical staff on dynamic resistance exercise for home-based rehabilitation and patient follow-up through phone calls. The home-based resistance intervention approach under the instruction of clinical staff was established on the basis of patients’ improved dynamic balance and physical function; CST, TUG, and WOMAC scores were significantly enhanced.

This study has some limitations that should be addressed. First, because we enrolled older participants who lived far from the medical center, it was difficult to perform exercises under the supervision of a physical therapist. However, participants were followed up by clinical staff through phone calls or using a communication application. The outcomes also showed promising effects. Second, we were required to administer isometric contraction exercises to the isometric group instead of the untreated control group. Third, changes in body composition, physical activity level outside, and dietary changes were not recorded, which could potentially have a bias. Fourth, based on the number of potentially eligible participants who were excluded, it appears that the generalisability of the results to all patients with T2DM and knee OA is relatively low. Fifth, there was no assessment of potential differences in external physical or occupational activities for the participants that might have influenced the results. Sixth, we did not record details of whether family members supervised the interventions, which may have led to bias in the study results.

We encourage older patients with comorbid T2DM and mild-to-moderate knee OA to commence a resistance exercise routine (5 sets of 10 reps 3 times a week for 12 weeks). We did not observe considerable changes in HbAlc level that corresponded to specific resistance exercise approaches; however, we revealed that in particular, muscle strength, dynamic balance, and physical function can be significantly improved by dynamic resistance exercise. In addition, the straightforward 10-rep exercise program is designed to train the major lower extremity muscles necessary for functional mobility (e.g., walking, getting into and out of a chair, walking up and down stairs). The 10-rep seated exercise can feasibly be performed at home, meaning it is safe for older patients. Future studies should investigate the long-term effects of this program on a larger population. In addition, the effects of combining aerobic and resistance exercises could be studied.

## 5. Conclusions

A 12-week dynamic resistance training program led to superior improvement in muscle strength, balance, and physical function compared with isometric exercise in older patients with T2DM and knee osteoarthritis. However, no notable difference regarding change in HbA1c level was observed among different resistance exercises. Considering the flexibility, safety, ease, and effectiveness of resistance training, health care professionals who treat this population should consider recommending a dynamic or isometric exercise regime delivered through a home-based rehabilitation program. However, the aforementioned limitations should be addressed.

## Figures and Tables

**Table 1:** Baseline characteristics of participants.

Characteristics	Dyn (n = 30)	Iso (n = 30)	*p*
Gender			
Male/female, n (%)	11 (37%)/19 (63%)	18 (60%)/12 (40%)	0.325
Age (year)	65.9 (2.9)	65.0 (3.1)	0.233
Body height (cm)	159.16 (9.75)	157.29 (7.30)	0.404
Body weight (kg)	67.65(12.02)	69.68(13.53)	0.542
Body mass index (kg/m^2^)	26.68 (4.17)	28.11 (4.93)	0.231
Duration of T2DM (year)	9.13 (7.40)	11.46 (7.77)	0.239
HbA1c (%)	7.24 (1.1)	7.43 (1.2)	0.522
Kellgren–Lawrence grade	1.66 (0.80)	1.47 (0.82)	0.343
I, n (%)	16 (53)	22 (73)	
II, n (%)	8 (27)	2 (7)	
III, n (%)	6 (20)	6 (20)	
Taking NSAIDs n (%)	1 (3.3%)	1 (3.3%)	1

Dyn = dynamic group, Iso = isometric group. T2DM = Type 2 diabetes mellitus. Values are means with standard deviations (in brackets) unless indicated otherwise. Non-steroidal anti-inflammatory drugs (NSAIDs).

**Table 2:** Improvements in outcomes after the 12-week intervention between groups.

Outcomes	Group	Baseline	Post-Intervention	Change (95% CI)	Effect Size (Cohen’s d)	Within-Group, *p*	Between Group, *p*	MDC
**HbA1c (%)**	Dyn	7.24 ± 1.14	7.06 ± 0.83	−0.18 (−0.47; −0.11)	0.18	0.212	0.462	N
	Iso	7.43 ± 1.17	7.24 ± 1.04	−0.19 (−0.55; 0.17)	0.17	0.29		N
**CST (reps) #**	Dyn	12.08 ± 2.39	13.38 ± 3.68	1.3 (0.46; 2.14)	0.42	0.004	0.011	Y
	Iso	10.55 ± 2.64	11.30 ± 2.33	0.75 (0.25; 1.25)	0.39	0.004		N
**TUG (sec) #**	Dyn	9.45 ± 1.73	8.25 ± 1.50	−1.2 (1.65; −0.74)	0.74	<0.001	<0.001	Y
	Iso	10.61 ± 1.69	9.8 ± 1.42	−0.82 (−1.31; −0.32)	0.51	0.002		N
				**WOMAC (score)**				
**Pain**	Dyn	2.93 ± 3.16	1.56 ± 2.12	−1.37 (−2.14; −0.61)	0.51	0.001	0.48	Y
	Iso	2.86 ± 2.40	1.93 ± 1.91	−0.93 (−1.54; 0.32)	0.43	0.004		N
**Stiffness**	Dyn	1.2±1.10	0.93 ± 0.83	−0.29 (-0.6; 0.1)	0.29	0.058	0.549	N
	Iso	1.1 ± 1.23	0.79±0.82	−0.31 (−0.77; 1.45)	0.29	0.174		N
**Physical Function #**	Dyn	7.67 ± 6.89	2.89 ± 3.27	−4.78 (−6.73; −2.84)	0.89	<0.001	0.033	Y
	Iso	7.93 ± 8.19	5.07 ± 5.47	−2.86 (−4.89; 0.84)	0.41	0.007		Y
**Total #**	Dyn	11.81 ± 10.32	5.37 ± 5.52	−6.44 (−8.97; -3.92)	0.78	<0.001	0.036	Y
	Iso	11.90 ± 11.21	7.79 ± 7.22	−4.11 (−6.79; 1.4)	0.43	0.004		Y

Dyn = dynamic group, Iso = isometric group; CST = 30-s chair stand test; TUG = 3-m timed up-and-go test; Changes indicates from baseline to post-intervention; &: nonsignifcant difference in all parameter at baseline # significant group main effect, *p* < 0.05; Y: exceeded MDC: minimum detectable change; N: Not exceeded MDC.
